# Temporal Gene Expression Analysis and RNA Silencing of Single and Multiple Members of Gene Family in the Lone Star Tick *Amblyomma americanum*

**DOI:** 10.1371/journal.pone.0147966

**Published:** 2016-02-12

**Authors:** Rebekah L. Bullard, Jaclyn Williams, Shahid Karim

**Affiliations:** Department of Biological Sciences, University of Southern Mississippi, Hattiesburg, MS, United States of America; Metabiota, UNITED STATES

## Abstract

Saliva is an integral factor in the feeding success of veterinary and medically important ticks. Therefore, the characterization of the proteins present in tick saliva is an important area of tick research. Here, we confirmed previously generated sialotranscriptome data using quantitative real-time PCR. The information obtained in this in-depth study of gene expression was used to measure the effects of metalloprotease gene silencing on tick feeding. We analyzed the temporal expression of seven housekeeping genes and 44 differentially expressed salivary molecules selected from a previously published *Amblyomma americanum* sialotranscriptome. Separate reference genes were selected for the salivary glands and midgut from among the seven housekeeping genes, to normalize the transcriptional expression of differentially expressed genes. The salivary gland reference gene, ubiquitin, was used to normalize the expression of 44 salivary genes. Unsurprisingly, each gene family was expressed throughout the blood meal, but the expression of specific genes differed at each time point. To further clarify the complex nature of the many proteins found in the saliva, we disrupted the translation of several members of the metalloprotease family. Intriguingly, the nucleotide sequence similarity of the reprolysin metalloprotease gene family is so homologous that a single synthesized dsRNA sequence knocked down multiple members of the family. The use of multigene knockdown yielded a more significant picture of the role of metalloproteases in tick feeding success, and changes were observed in the female engorgement weight and larval hatching success. Interestingly, the depletion of metalloprotease transcripts also reduced the total number of bacteria present in the salivary glands. These data provide insight into the expression and functions of tick salivary proteins expressed while feeding on its host.

## Introduction

*Amblyomma americanum*, the Lone Star Tick, is widespread across the entire eastern seaboard of the USA and westward as far as central Texas, and it has begun to invade the central plains [1–6]. The geographic distribution of *A*. *americanum* has probably increased through its association with the white-tailed deer (*Odocoileus virginianus*), its keystone host. However, the Lone Star Tick has been found on a wide variety of hosts, including humans, cattle, horses, dogs, and cats [6–8]. The nonspecific and aggressive nature of *A*. *americanum* is very important to the veterinary and medical communities, as it is a vector of diseases such as spotted fever group rickettsiosis, human monocytic ehrlichiosis, southern-tick-associated rash illness, theileriosis, tularemia, *Heartland* virus infection, and infection with *Tacaribe* virus, newly discovered in this tick (for a review, see [1,7–10]). In addition to these diseases, *A*. *americanum* has been associated with delayed anaphylaxis to red meat and is the first recorded example of an ectoparasite causing food allergy [11,12].

Tick feeding requires the insertion of the hypostome into the host’s skin and the formation of a blood pool beneath the dermis at the bite site. This blood pool remains fluid throughout tick feeding, which may take several days, during which the female will become engorged and grow to more than 100 times her original mass [13]. To maintain the blood pool, the tick salivary glands secrete an abundance of pharmacologically active compounds into the host through the saliva [14]. Most piercing injuries, similar to tick bites, would elicit such a strong inflammatory response and hemostasis that the host would be extremely aware of the foreign object. However, ticks must attach to their hosts for many days or weeks (depending on the species) to ingest a full blood meal. The protein composition of tick saliva has been examined in the sialomes of many species. The molecules and proteins secreted into the host through the saliva are responsible for: 1) preventing clot formation, to maintain the blood pool; 2) blocking the host immune signaling molecules, to prevent immune cascades; 3) preventing the host inflammatory response, to reduce swelling, erythema, and localized pyrexia; and 4) the transmission of pathogens from the tick salivary glands to the host [14]. The proteins produced by the salivary glands that are secreted into the host through the saliva come into direct contact with the host and have the greatest effect of any proteins on tick feeding. Although similar proteins are found in the midgut, focusing on the salivary proteins increases our understanding of how the tick interacts with its host. Tick saliva contains many redundant proteins, including multiprotein families that are differentially regulated, suggesting that they play roles in the evasion of the immune defenses of the vertebrate host [15]. Many researchers have examined the clinical applications of these tick proteins because of their potential utility in the treatment of medical conditions, such as autoimmune diseases [16], vascular dysfunction [17], and blood clotting disorders [18]. However, even with increasing access to “big data”, these biomolecules require further functional characterization.

Sialotranscriptomes are currently the standard technique used to identify the proteins predicted to occur in the salivary glands of ticks. This technique uses cDNA isolated from the salivary glands of unfed, partially fed, and/or fully fed females as sequencing templates. The investigation of several feeding points allows any transcriptional variation to be detected. However, it is important to confirm these data with a more targeted approach, such as quantitative real-time PCR (qPCR). Because this housekeeping gene plays a central role in the determination of transcriptional gene expression, it is vitally important to select an appropriate stably expressed gene. The optimal housekeeping gene used as a reference can differ between tick species and tick tissues. A study of the various life stages of *Rhipicephalus microplus* and *R*. *appendiculatus* revealed that elongation factor 1A was the most suitable reference gene when two expression-normalization algorithms were compared (geNorm and NormFinder) [19]. Although the geNorm analysis showed less variation among the six reference genes, the amount of sample required to examine the expression of these six genes is prohibitive in tick research. A similar study of *Ixodes scapularis* examined the expression levels of multiple housekeeping genes in two different tissues throughout the blood meal [20]. The expression of the selected housekeeping genes within the *I*. *scapularis* tissues showed little variation throughout the blood meal, and two ribosomal proteins varied least in both the synganglia and the salivary glands. A previous study from our research group of the Gulf Coast tick, *A*. *maculatum*, showed that actin is the most suitable gene for normalizing the expression of other genes across the tick life stages and throughout the adult blood meal [21]. That study also compared the expression levels of a gene of interest, *VAMP ½*, using each of several tested housekeeping genes. It was found that the calculated expression of *VAMP ½* varied greatly (up to 70%) depending on the housekeeping gene used [21]. The variation in the effects of the housekeeping gene used to normalize gene expression simply among Ixodidae tick species demonstrates that the reference gene used must be validated in each individual tick species. Here, we evaluated the transcriptional stability of seven housekeeping genes throughout the blood meal in two separate tissues (salivary gland and midgut). These data confirm the need for reference gene verification in individual tissue types.

The *A*. *americanum* sialotranscriptome [22] was used to identify the multiple genes that are differentially expressed throughout the blood meal. The work presented here confirms the expression measured with RNA-Seq and examines in more detail the wide ranging transcription of selected genes throughout the blood meal. The expression of these genes was assessed at eight feeding time points (unfed, 24, 48, 72, 96, 120, 144, and 168 h postinfestation) to determine the expression levels of individual genes in the salivary glands of female *A*. *americanum*. The identification of five reprolysin genes, with 75%–85% nucleotide identity, allowed us to silence multiple genes from the same family to clarify the role of this family in tick blood feeding. In addition to a phenotypic analysis of the reprolysin-knockdown ticks, the microbial load was measured with 16S ribosomal RNA (rRNA) qPCR amplification. Further screening with RNA interference (RNAi) of the tick salivary protein families will extend our understanding of their functional roles and allow the identification and development of antitick molecules.

## Results and Discussion

### Reference Genes Differ by Tissue Type

The first aim of this work was to determine the suitability and stability of various housekeeping genes in the tick salivary glands and midgut for use in the normalization of transcriptional gene expression. Other studies have usually examined expression stability throughout all tissues at a single time point or compared it in multiple tissue types at multiple time points [19–21]. Here, the salivary glands and midgut were analyzed separately to identify any differences in the time-dependent profiles of housekeeping gene expression during blood feeding. Differences in the expression of a housekeeping gene are probably attributable to its tissue-specific expression, based on differences in the cellular organization and cell types of the tissues [20].

NormFinder requires a standard curve to calculate the number of transcript copies in each sample at each time point [23]. The number of copies of each transcript throughout the blood meal is then used to calculate a stability value. The stability value and the suitability as a reference gene are inversely related. BestKeeper, unlike NormFinder, uses raw data rather than relative quantities. BestKeeper identifies the most stable reference gene as the gene with the lowest standard deviation (SD) and the highest coefficient of correlation with the BestKeeper index (which uses the geometric mean of the candidate C_t_ value) [24]. The ΔΔCt method compares the Ct values of the tested sample against a reference [25]. In this study, unfed tissue samples were used as the reference against which the remaining samples were compared. The use of unfed tissues as the reference gives a baseline of expression before stressors are applied or metabolism is increased by feeding. After ΔΔCt is calculated, the values can be converted into the amount of target transcript with the following equation:

Amountoftarget=2(−ΔΔCt)

**Fig 1:**
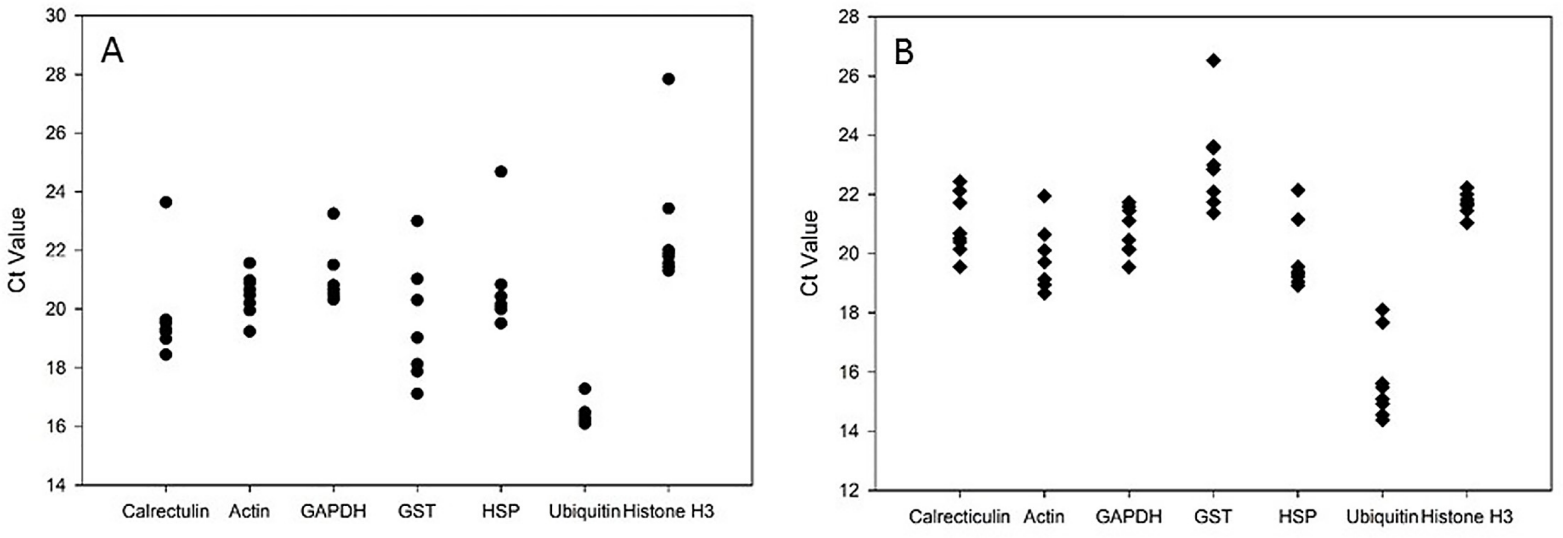
Temporal expression of housekeeping genes. Raw Ct values for the mRNA of seven *A*. *americanum* housekeeping genes from tick salivary glands (A) and midgut (B) throughout a blood meal (unfed–168 h). Each gene was amplified from each sample and the point at which the amplification curve crosses the threshold is considered the Ct.

After the amount of target is determined, the SD can be calculated. The sample with the smallest SD is the gene with the most consistent expression levels. Each gene was ranked based on its suitability as a reference gene, as shown in S1 and S2 Tables for the salivary glands and midgut, respectively. Ubiquitin in the salivary gland samples produced Ct values with the least variation (Fig 1) and was ranked first according to BestKeeper and the ΔΔCt method. When the methods described above were used, histone H3 was the most stable transcript in the midgut samples according to BestKeeper and the ΔΔCt method. It is important to note that the genes tested displayed different expression profiles in each tissue. In previous studies, optimal reference genes were identified by examining the expression of a transcript in combined tissue types at different time points, whereas the results presented here indicate that reference genes should be selected separately for each tissue type. In light of these results, qPCR results may be best normalized when the normalization reference are selected based on tissue type and developmental stage.

### Temporal Analysis of *A*. *americanum* Gene Expression

After the most stable reference gene for the *A*. *americanum* salivary glands was determined, the temporal gene expression of 44 salivary genes was measured. The work presented here serves to validate the data collected with RNA-Seq [22] and extends that data by measuring the expression of these genes during each 24 h time period, rather than across combined time points. Some *A*. *americanum* genes were not amplified at any specific time point, which may be attributable to the folding and protection of the mRNA or to primer inefficiency. This may arise from inefficient primer binding if the sequences formed secondary structures, preventing proper amplification. It is also possible that differences in the feeding seasons in which the samples were collected affected some genes expressed during only one of the collection times.

#### Protease inhibitor domain

**Fig 2:**
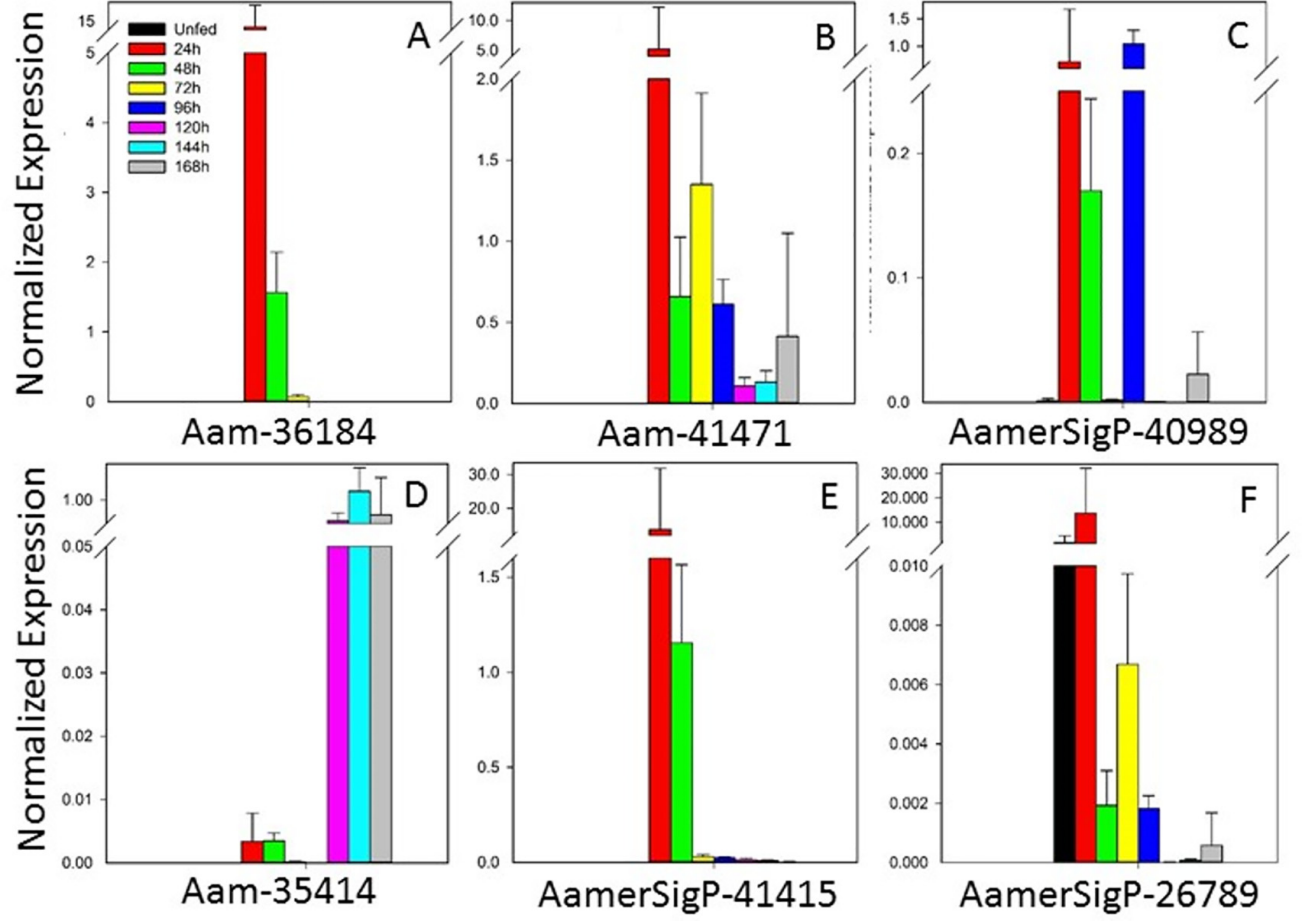
Temporal expression of genes encoding protease inhibitor domains. Six genes from two protein families are shown.

Two protein families that contain protease inhibitor domains were selected from the sialotranscriptome for further analysis (Fig 2). Four genes that contain at least one Kunitz protease inhibitor domain showed a range of expression profiles. Gene Aam-36184 (Fig 2A) was most strongly expressed before the 48 h time point, as was Aam-41471 (Fig 2B), albeit at a lower level. Gene AamerSigP-40989 (Fig 2C) displayed a unique expression profile throughout the blood meal, with its expression occurring specifically at 24–48 h and 96 h. Last in the Kunitz superfamily genes, Aam-35414 (Fig 2D) was differentially expressed during the later time points of tick feeding, with highest expression in the period 120–168 h.

Kunitz-domain proteins are one of the protease inhibitor families most frequently detected in sialome studies [26,27]. Kunitz-containing peptides have been described in several ticks, including *I*. *scapularis*, *R*. *appendiculatus*, and *Ornithodoros moubata* [28,29]. The Kunitz-containing peptides of *I*. *scapularis* are closely related to tick-derived protease inhibitor (TdPI), a strong inhibitor of human skin β-tryptase [29]. About 15 single-Kunitz-domain peptides from ticks have been functionally characterized in the literature. These proteins vary in the number of cysteine motifs (normally about six Cys residues), and in *I*. *scapularis*, these proteins are organized into three groups (I, II, and III) based on their Cys motifs [30].

An *I*. *scapularis* protein, Ixolaris, has been recombinantly expressed and is a known to inhibit Factor VIIa [31]. This effectively inhibits the blood coagulation pathway, which benefits the tick in allowing the formation of the liquid blood pool. In *Haemaphysalis longicornis*, a unique Kunitz-domain-containing inhibitor, haemaphysalin, is responsible for interrupting the kallikrein–kinin system [32]. This system induces the production of additional blood coagulation compounds and members of the inflammatory pathway. The involvement of Kunitz-domain-containing protease inhibitors on a known inflammatory signaling system suggests that the protein family functions in other aspects of tick feeding.

These peptides have bacteriostatic activity in the tick *Dermacentor variablis*. A Kunitz-type serine protease inhibitor can limit rickettsial colonization within the midgut [33,34]. A Kunitz-type serine protease inhibitor is also differentially expressed in *R*. *microplus* in an ovarian response to *Babesia bovis* infection [35].

The second family selected for further analysis included proteins containing trypsin-inhibitor-like domains (TIL II domains). These two genes (AamerSigP-41415 and AamerSipP-26789; Fig 2E and 2F) are both expressed before 48 h and the highest levels of expression were detected at 24 h after attachment. The TIL proteins typically have five disulfides bonds, formed by 10 cysteines [36]. Recently, a subtilisin inhibitor with a TIL II domain identified in *R*. *microplus* displayed antimicrobial properties, suggesting that these polypeptides may also have a role in mediating microbial loads [37].

#### Lipocalins

**Fig 3:**
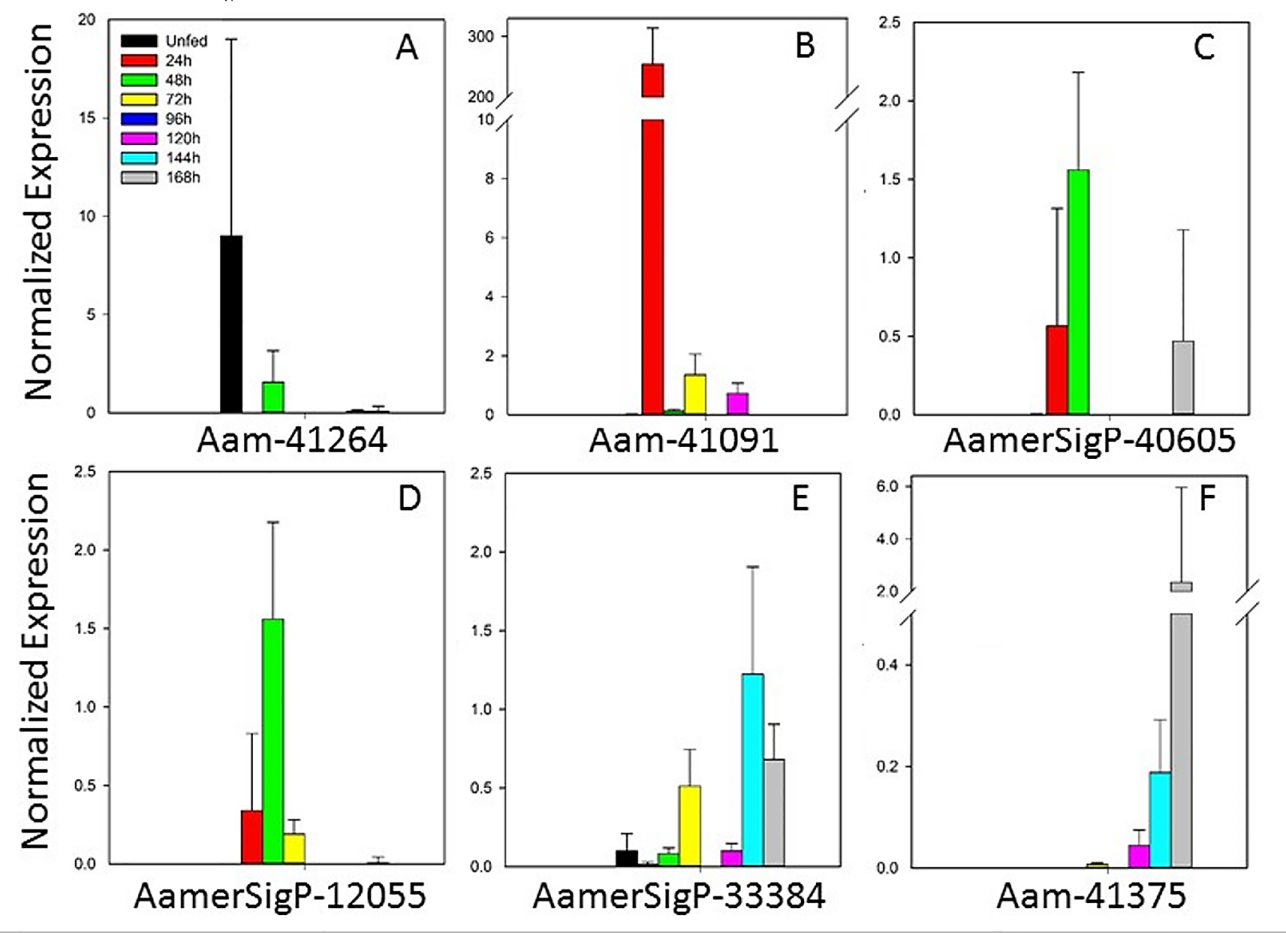
Temporal expression of lipocalin genes. AamerSigP-18604 was not expressed at any time point, according to qRT–PCR.

The expression of seven lipocalin genes suggests that this family plays vital roles throughout the blood meal (Fig 3). Aam-41264, Aam41091, AamerSigP-40605, and AamerSigP-12055 (Fig 3A–3D) were more strongly expressed between the unfed and 48 h time points, whereas AamerSigP-33384 and Aam-41375 were more strongly expressed during the later time points (Fig 3E and 3F). Interestingly, gene AamerSigP-18604 was not expressed throughout the blood meal, according to qRT–PCR (data not shown).

The lipocalin proteins in tick saliva are believed to play a role in the immune response and cell homeostasis. Although lipocalins may not play a direct role in the initial feeding mechanism of the tick, they are vitally important in the ability of the tick to avoid detection by the host’s immune system [38,39] and to remain attached for the duration of the blood meal. They are often characterized by diverse functions and low molecular weights [40]. As a whole, the family has little sequence identity, but conserved motifs occur throughout the sequences, and are used to identify members of the family. Lipocalins characteristically contain an eight-stranded antiparallel β-sheet, which forms a β-barrel. These proteins have previously been identified in other soft and hard ticks, including *Ornithodoros moubata* [41], *R*. *appendiculatus* [42], and *D*. *reticulatus* [43]. A study of lipocalins in male and female *I*. *ricinus* ticks showed similar variations in their expression between the sexes and feeding stages [44]. Lipocalins reduce the host histamine at the feeding site and reduce or prevent the histamine-mediated cutaneous inflammation response of the host. Lipocalins also have other well-characterized functions, including ligand binding (retinoids and various steroids), receptor binding (possible transport mechanism for steroids), and pheromone activities (odorant-binding properties).

#### Glycine-rich proteins

**Fig 4:**
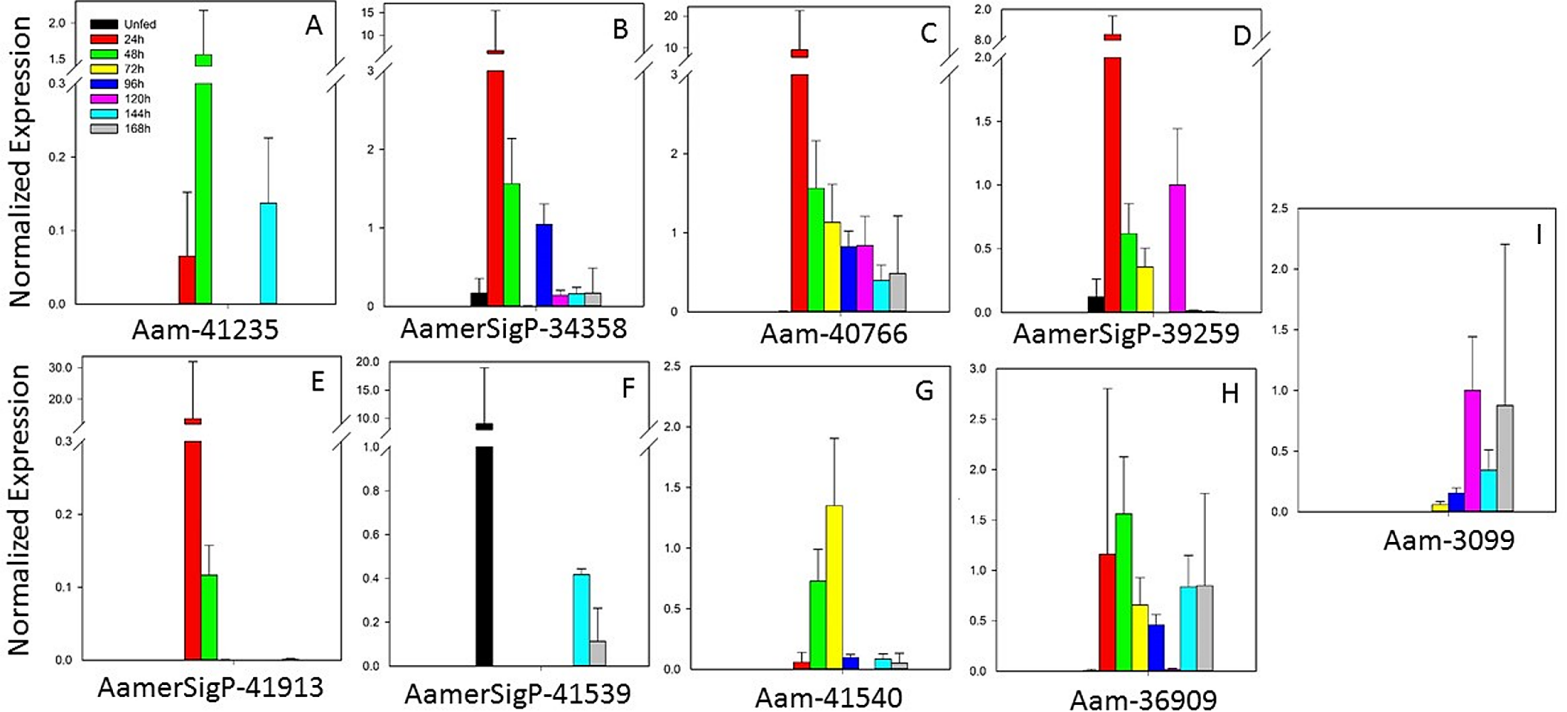
Temporal expression of glycine-rich protein genes.

The glycine-rich protein (GRP) family is a diverse class of proteins characterized only by the predominance of glycine residues. Because of the variability in this class of proteins, the functions of many GRPs have not been identified. The expression profiles of nine GRPs showed a range of differential expression throughout the blood meal (Fig 4). Gene Aam-41235 (Fig 4A) was expressed at the 48 h time point, with little or no expression at the other time points. AamerSigP-34358, Aam-40766, and AamerSigP-39259 (Fig 4B–4D) showed the highest levels of expression at 24 h after attachment, which tapered off throughout the remaining blood meal. After 96 h, AamerSigP-34358 expression levels decreased dramatically, whereas Aam-40766 expression continued to taper off from the 24 h level. The early expression of AamerSigP-41913 (Fig 4E) was similar to that of the other genes, but became minimal after 24 h. AamerSigP-41539 (Fig 4F) was unique among the GRPs selected for this study in that it was almost exclusively expressed in the unfed salivary glands. Aam-41540 (Fig 4G) also showed a distinctive profile, being expressed primarily between 48 h and 72 h. Aam-36909 and Aam-3099 (Fig 4H and 4I) displayed different profiles (consistently expressed and late expression, respectively), but the transcript levels of Aam-41540, Aam-3099, and Aam-36909 remained centered around 1.0 normalized expression unit, indicating that none of them was significantly differentially expressed during feeding.

Because little information is available on the functions of GRPs, a wide range of expression profiles was not unexpected. Members of the GRP family characterized based on function, so multiple functions could be represented in the few GRPs selected here. Without additional information on the functions of these proteins, it is difficult to compare the gene expression profiles of members from the same class. Different functions during the blood meal could explain the different expression profiles. It is also possible that some of the genes selected here are members of the same class and have similar or redundant functions.

A significant proportion of GRP research has been undertaken in spiders and plants. In plants, GRPs have been characterized by the presence of glycine repeats and conserved domains within the protein. The repeats characterized in plants include GGX, GGXXXGG, GXGX, general glycine rich, and GGX/GXGX [45]. Plant GRPs are also characterized by their involvement in the cell wall, RNA binding, and cold shock [46]. However, little is known about their functions in ticks, so their classification generally involves the identification of glycine repeats.

GRPs may perform many functions in ticks, but they are typically attributed to the formation of a proteinaceous matrix around the tick mouthparts, known as the ‘cement cone’. This cone is believed to help anchor the tick into the host dermis and to protect the mouthparts from the host’s immune responses. The firm attachment of the tick to the host allows its uninterrupted access to the blood meal. The vaccine potential of two GRPs has been investigated. In a study of 64P, a GRP found in *R*. *appendiculatus*, antibody staining was used to verify the presence of 64P within the cement cones embedded in hamster skin. That study also examined the expression of 64P during blood feeding, which was highest in the first three days of feeding, after which it returned to the unfed levels or lower [47]. Research into a second GRP in the same species, RIM36, examined the antigenicity of the protein against antisera collected from tick-infested cattle. Using antiserum, RIM36 was localized to the salivary glands and was also identified in the cement lysate, verifying its role in the cement structure [48].

#### Tick-specific family: unknown functions

**Fig 5:**
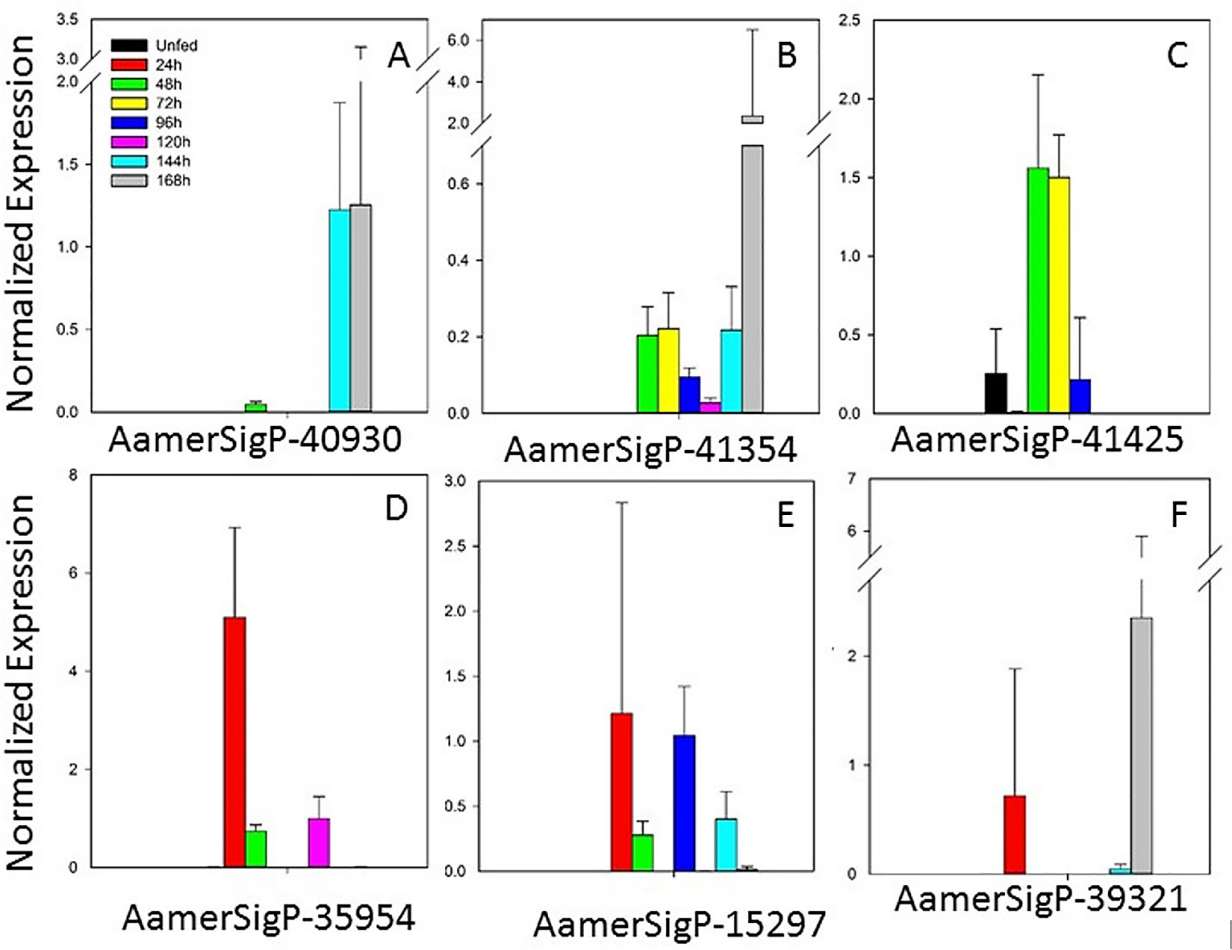
Temporal expression of seven tick-specific genes with unknown functions. AamerSigp-22563 was not expressed, according to qRT–PCR.

Proteins belonging to this family are homologous to other putative tick proteins, although no function has yet been identified. The expression of these genes is sporadic, both for individual genes and for the whole family (Fig 5). Two genes (AamerSigP-40930 and AamerSigP-41354 Fig 5A and 5B) showed higher expression during the late feeding time points, which could indicate functions specific to the late feeding mechanisms or antigenic variation in a conserved function. AamerSigP-41425 (Fig 5C) is primarily expressed during the middle time points, 48–72 h. During this time, the tick prepares for the fast-feeding stage. Three genes (AamerSigP35954, AamerSigP-15297, and AamerSigP-39321; Fig 5D–5F) displayed sporadic expression throughout the course of the blood meal. Gene AamerSigP-22563 was not expressed at all, according to qRT–PCR (data not shown).

#### 8.9-kDa family

This protein family is characterized by the presence of an 8.9-kDa domain within the protein structure. Genes AamerSigP-40930, AamerSigP-41425, and AamerSigP-22563 represented the 8.9-kDa family in this study. This family of proteins is often divided into three groups, based on the commonality of sequences: 1) prostriates and metastriates; 2) metastriates only; and 3) more than one 8.9-kDa domain within the structure [15]. No functional role has been attributed to this class of proteins.

#### Ixoderin family

This family of proteins is represented by genes AamerSigP-35954 and AamerSigP-15297. Ixoderins, also known as ficolins, are oligomeric lectins that have both collagen- and fibrinogen-like domains. They are often considered to be binding complexes for viral, bacterial, and protozoan pathogens (specifically the glycosylated components of pathogens), activating complement molecules via the lectin pathway, and are therefore understood to be important in innate immunity [49,50]. Lectins have previously been found in soft ticks as membrane and soluble proteins, and their coding sequences have high homology to those of the ixoderins [50]. Although the exact function of the ixoderins in ticks is unknown, invertebrate and arthropod lectins are considered analogues of the immunoglobulins and tick lectins have been implicated in the transmission of pathogens to the host [51]. Because of differences in sample collection of different studies (salivary gland tissues versus saliva), it is possible that these molecules are used by the salivary glands themselves and are not secreted into the host. Their inferred functions in the tick innate immune response would facilitate tick feeding by reducing the pathogen load within the tick and therefore in the saliva. This reduction in pathogen load would reduce the immunological assault on the host’s immune system. However, this possibility has not been explored experimentally.

#### Immunity-related proteins

**Fig 6:**
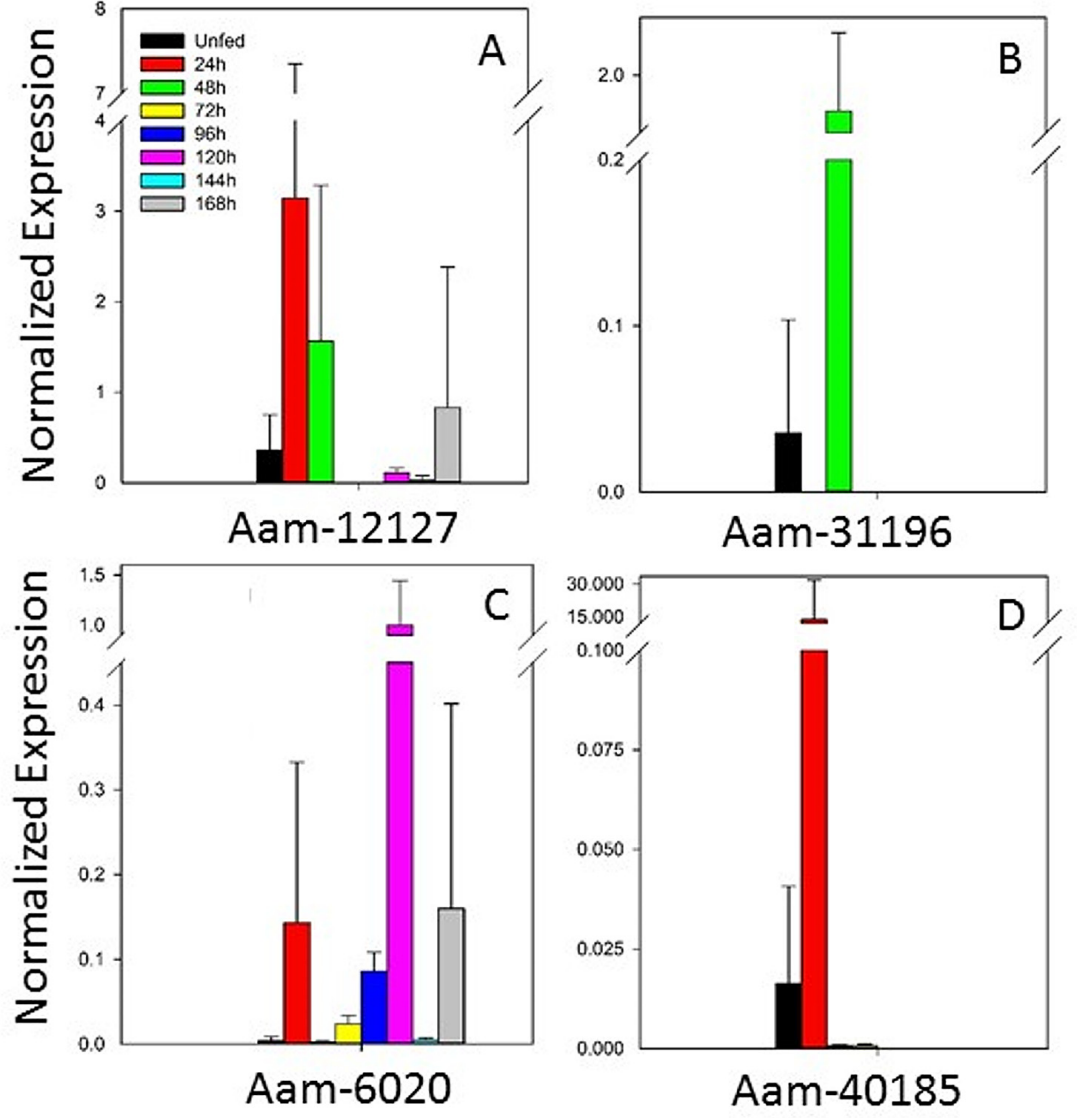
Temporal expression of immunity-related genes. Notably, gene AamerSigP-41992 was not expressed, according to qRT–PCR.

Other proteins selected from the transcriptome for investigation included those involved in tick immunity. The expression of an evasin gene, Aam-12127 (Fig 6A), was highest before 48 h of feeding. Another evasin gene, AamerSigP-41992, showed no expression according to qRT–PCR (data not shown). Evasins bind chemokines, particularly those involved in the proinflammatory responses, to avert the host immune response. The specificity of some of the evasin group has been clarified with cross-linking assays. Evasin-1 inhibits the binding of CCL3 and CCL4 to their respective receptors [51], whereas evasin-3 inhibits the binding of CXCL8 to its receptor CXCR1 and prevents neutrophil influx during an inflammatory event [52]. The inability of host chemokines to bind to their receptors prevents the host from mounting a proinflammatory response against the tick bite. It has also been shown that different species maintain distinct chemokine-binding profiles. This may be attributable, in part, to the size of the tick mouthparts and its engorgement weight. A study of *A*. *variegatum*, *I*. *ricinus*, *D*. *reticulatus*, and *R*. *appendiculatus* measured the reactivity of tick nymphal proteins to human and murine chemokines. The study showed that *A*. *variegatum* protein extracts contained the largest repertoire of chemokine-binding molecules, possibly because its larger mouthparts cause more extensive damage to the host [53].

Together with evasins, another immunity-related peptide, defensin, was tested (Fig 6B). This gene, Aam-31196, was primarily expressed at 48 h during the blood meal. Defensins are the most well-defined class of antimicrobial peptides in arthropods. Defensin proteins are typically produced as preproproteins [54], and the mature peptide is translated together with two extra sequences that are cleaved off at different points in the maturation process. Defensins are small peptides with typical mature molecular weights of < 9 kDa. The homology within the defensin class is low, but a conserved pattern of cysteines (typically six in insects) is necessary for proper protein folding.

Insect defensins have been identified in many different classes of insects. These proteins are primarily active against Gram-positive bacteria [55]. Defensins are expressed in all tick tissues, but the defensins that localize only in the salivary glands, such as DefMT2 and DefMT5 of *I*. *ricinus* [56], are likely secreted into the host through the saliva. They may assist tick feeding by reducing the microbial contamination at the puncture site, thus reducing the host immune response. Genes Aam-6020 and Aam-40185 (Fig 6C and 6D), which belong to the 5.3-kDa peptide family, are also listed as encoding immunity-related peptides.

#### Miscellaneous proteins

**Fig 7:**
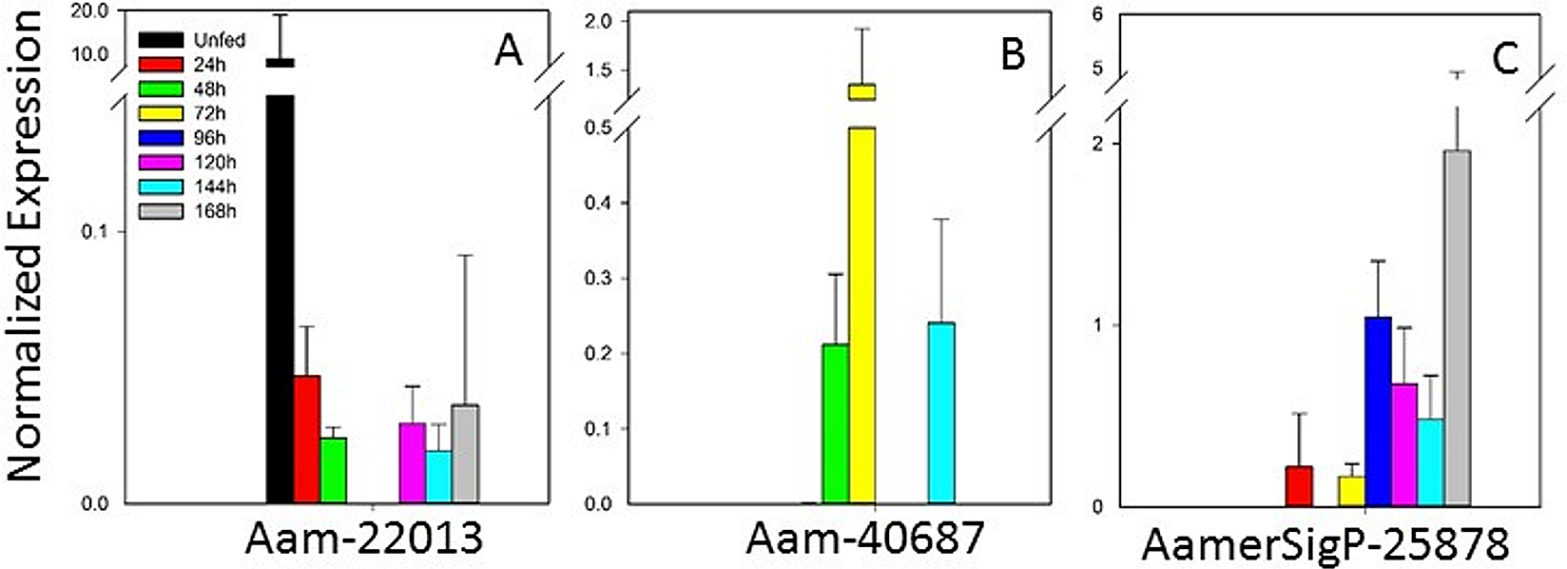
Temporal gene expression of a miscellaneous pool of protein families. Aam-5252 was not expressed, according to qRT–PCR.

This group of genes encodes a variety of protein families, including novel salivary proteins, cytoskeletal proteins, protein modification, and extracellular matrix proteins (Fig 7). As noted in the families discussed above, no expression of one gene (Aam-5252) was detected with qRT–PCR (data not shown). The remaining genes showed a range of expression profiles, including early expression (Aam-22013; Fig 7A), mid-point expression (Am-40687; Fig 7B), and late expression (AmerSigP-25878; Fig 7C).

#### Metalloprotease family

**Fig 8:**
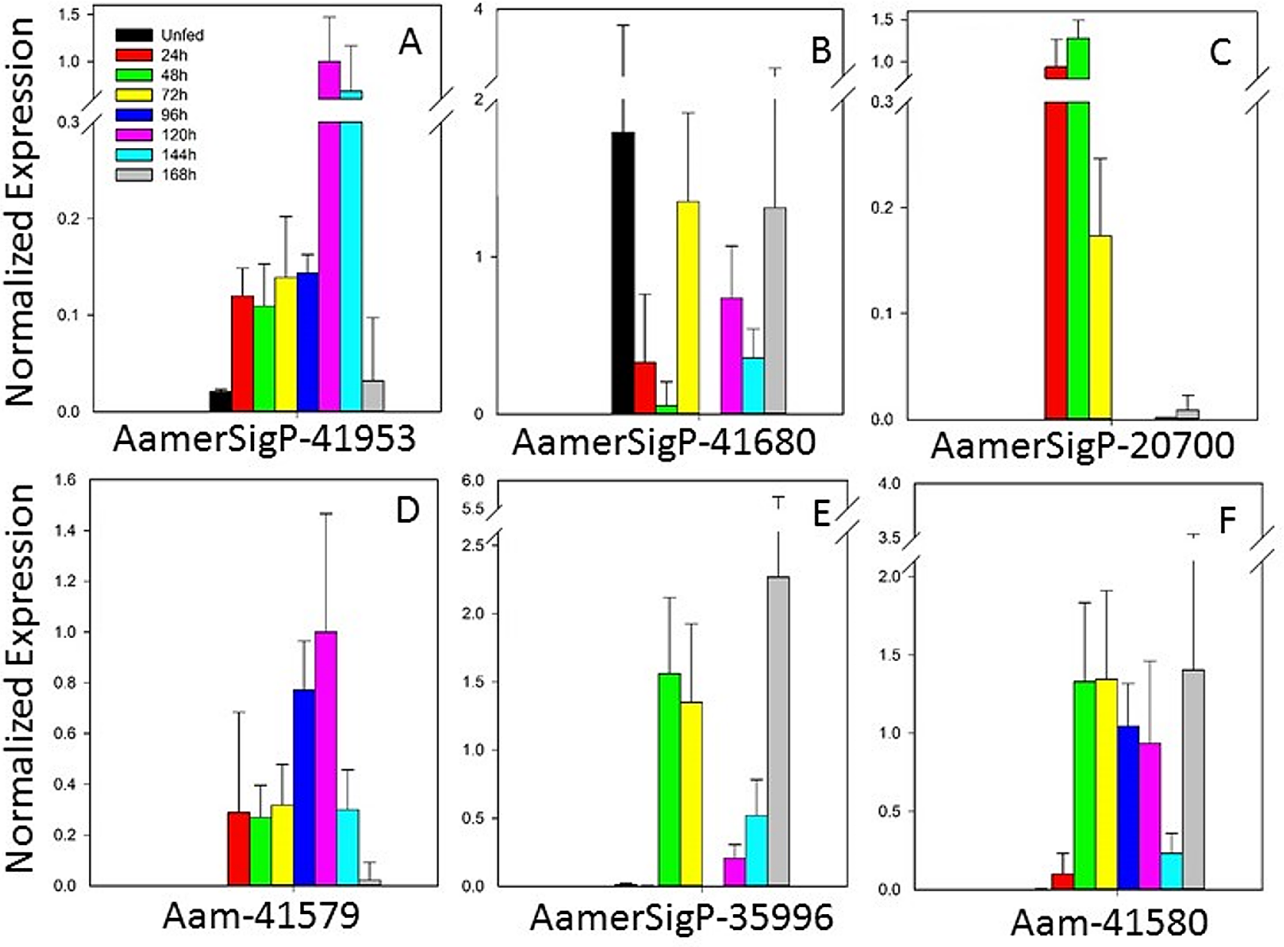
Temporal expression of seven metalloprotease genes encoding two protein families, measured with qRT–PCR.

The metalloproteases (MPs) selected from the sialotranscriptome can be further divided into neprolysin-like and reprolysin-like (members of the M13 family) proteins (Fig 8). A single gene of the neprolysin-like class was selected from the sialotranscriptome, AamerSigP-41953 (Fig 8A). This gene showed highest levels of expression at 120 and 144 h. The functions of the neprolysin-like metalloproteases have previously been associated with the degradation of bioactive peptides in mammalian systems, including peptides with a role in the catabolism of proinflammatory peptides such as tachykinins and the atrial natriuretic peptide family [57]. This has been established in neprolysin knockout mice, which displayed peripheral hyperalgesia rather than analgesia [58]. Tick saliva has been shown to degrade bradykinin [59], a proinflammatory peptide. Genes encoding neprolysin-like MPs are abundant in the genomes of both *Drosophila melanogaster* and *Caenorhabditis elegans* (reviewed in [60,61]). Neprolysin and neprolysin-like peptides also play roles in the reproductive health of the invertebrates [60].

Within the reprolysin-like class, AamerSigP-41680 (Fig 8B) was expressed throughout the course of the blood meal, and AamerSigP-20700 (Fig 8C) expression was highest early in tick feeding (24–48 h). The other three reprolysin genes (Aam-41579, AamerSigP-35996, and Aam-41580; Fig 8D–8F) were more strongly expressed after 48 h and throughout the remainder of the feeding period. These expression profiles indicate that the reprolysin MPs are abundantly expressed as a family in the salivary glands during the entire blood meal, although different genes were expressed at different points of the blood meal. Analysis of the sequences obtained with RNA-seq demonstrates that the proteins contain theoretical signal peptides, suggesting that they are secreted into the tick saliva and act on components of the host extracellular matrix [62,63].

**Fig 9:**
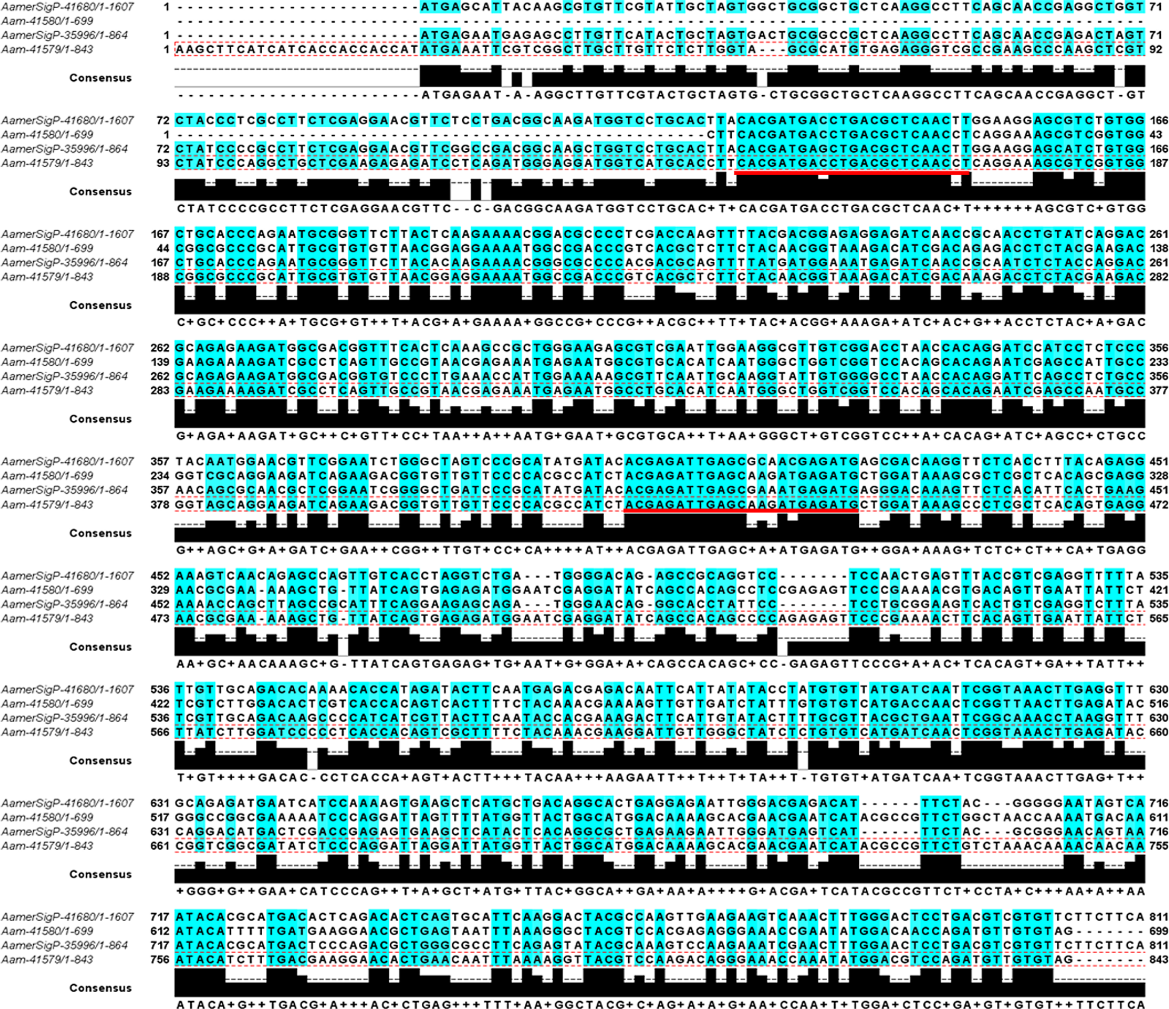
Multiple sequence alignment of reprolysin MP nucleotide sequences identified for multiple-gene RNAi. Knockdown was observed in four MPs. Nucleotides with more than 60% homology are highlighted in blue. Red underlining shows the locations of the primers. Actual primer sequences are listed in S3 Table.

Analysis of the five selected reprolysin nucleotide sequences showed high sequence similarity throughout much of the Aam-41579, Aam-41580, AamerSigP-35996, and AamerSigP-41680 genes (Fig 9). Although Aam-41579 had no signal peptide in its annotation, a later analysis identified a signal peptide (SignalP 4.1. Server). These metalloproteases are members of a Zn^2+^-dependent family of enzymes, secreted as proenzymes that require activation to become proteolytically active [57]. Each member of the family contains the zinc-binding motif HEXXHXXGXXHD. Other hard tick species also contain genes encoding metalloproteases, including *I*. *scapularis* [26] and *H*. *longicornis* [64]. Tick MPs have been associated with fibrinolytic and gelatinase activities, and the disaggregation of platelets [62,65]. The ability of tick reprolysin metalloproteases to degrade fibrin and fibrinogen demonstrates their roles in maintaining the blood pool [62,66]. All these functions have been associated with the tick’s ability to maintain a feeding cavity and suggest that these MPs interact directly with components of the host extracellular matrix [64,67]. Ticks are not alone in their use of MPs to interfere with blood clotting. Both snake and spider venoms contain MPs with activities that disturb homeostasis [68], which are the targets of snake venom antisera. Metalloproteases have also been associated with the dissemination of *Borrelia* spirochetes in human cells, because both matrix metallopeptidase 9 (MMP-9) and MMP-1 are upregulated in infected cells [69]. *Borrelia* spirochetes upregulate matrix MPs in human tissues to increase their movement across the tissues, linking the roles of MPs to pathogen transmission [69].

### Verification of Reprolysin Metalloprotease Knockdown in Adult Females

The metalloprotease gene Aam-41580 was selected for knockdown because it was highly expressed across many time points during the blood meal. The 95% depletion of the Aam-41580 transcripts caused a statistically significant reduction in tick weights relative to that of the noninjected controls. However, when compared with the controls injected with nonspecific dsRNA, there was insufficient data to clarify the role played by MP in tick feeding. It should be noted that the high levels of sequence homology between genes Aam-41580 and Aam-41579 caused both genes to be knocked down.

**Fig 10:**
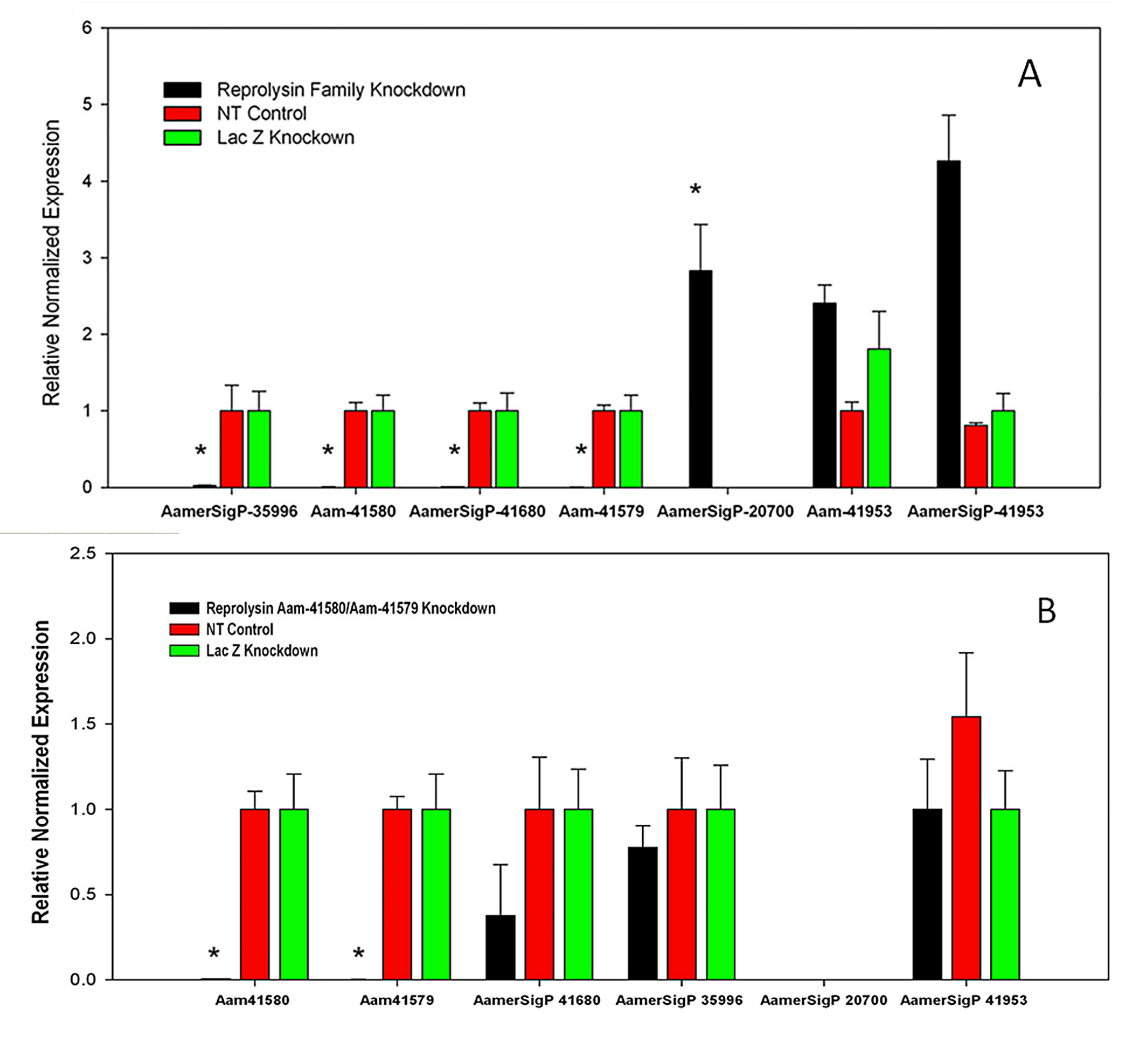
RNA interference (RNAi)-mediated silencing of preprolysin metalloproteases. (A) With RNAi, all members of a family of MP genes (Aam-41580, Aam-41579, AamerSigP-41680, and AamerSigP-35996) were successfully knocked down. Compensatory mechanisms were detected in response, with a three-fold up regulation of AamerSigP-20700 and a three-fold upregulation of the neprolysin AamerSigP-41953 gene. (B) Single RNAi Aam-41580 also resulted in a statistically significant reduction in Aam-41579 expression. No statistically significant compensatory mechanisms are shown. All knockdowns were 95% or more and * indicates P < 0.05 (statistically significant).

**Fig 11:**
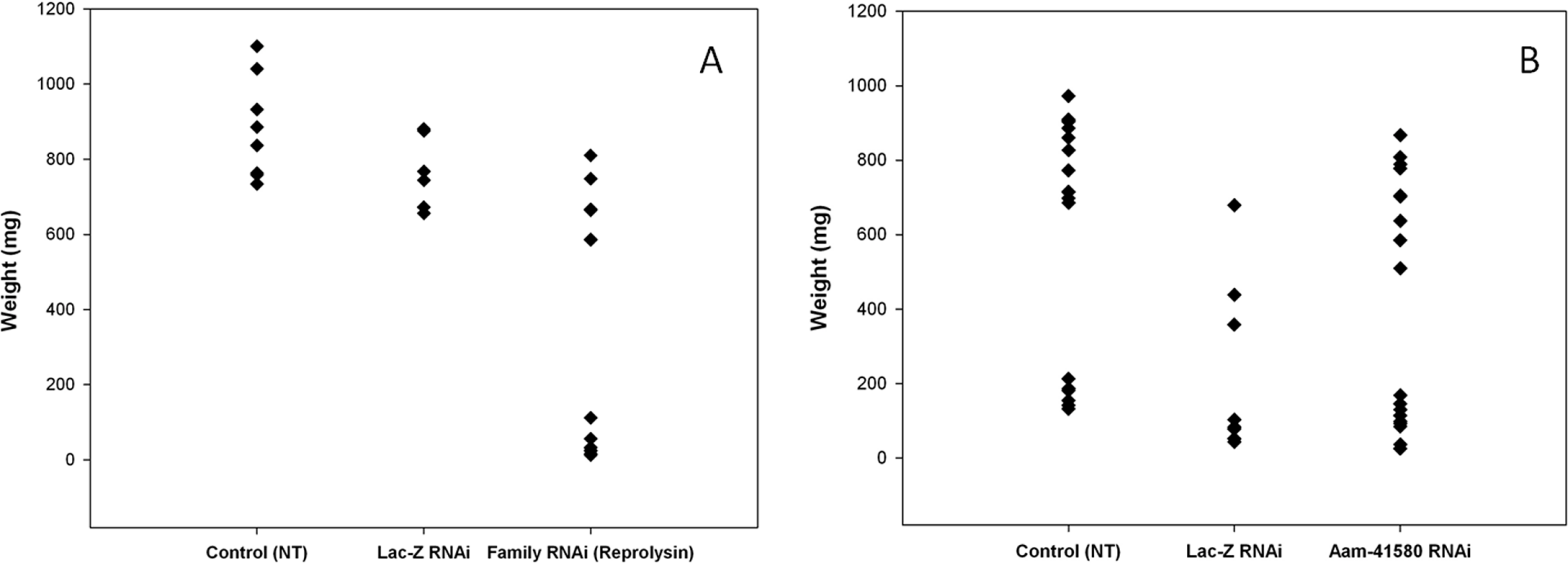
Engorgement weights of female ticks. Tick engorgement weights (mg) from both Rep-MP RNAi and Aa-41580 RNAi ticks fed on sheep. Am-41580 RNAi knockdown caused a significant reduction in tick weights (P < 0.05) compared with the (No Treatment) control; weights were measured on day 10. The size of the ds-Lac-Z (irrelevant RNA)-treated sample was significantly reduced during feeding and was not included in the analysis. RNAi knockdown of the reprolysin family significantly reduced tick weight (P < 0.05) compared with both controls.

**Fig 12:**
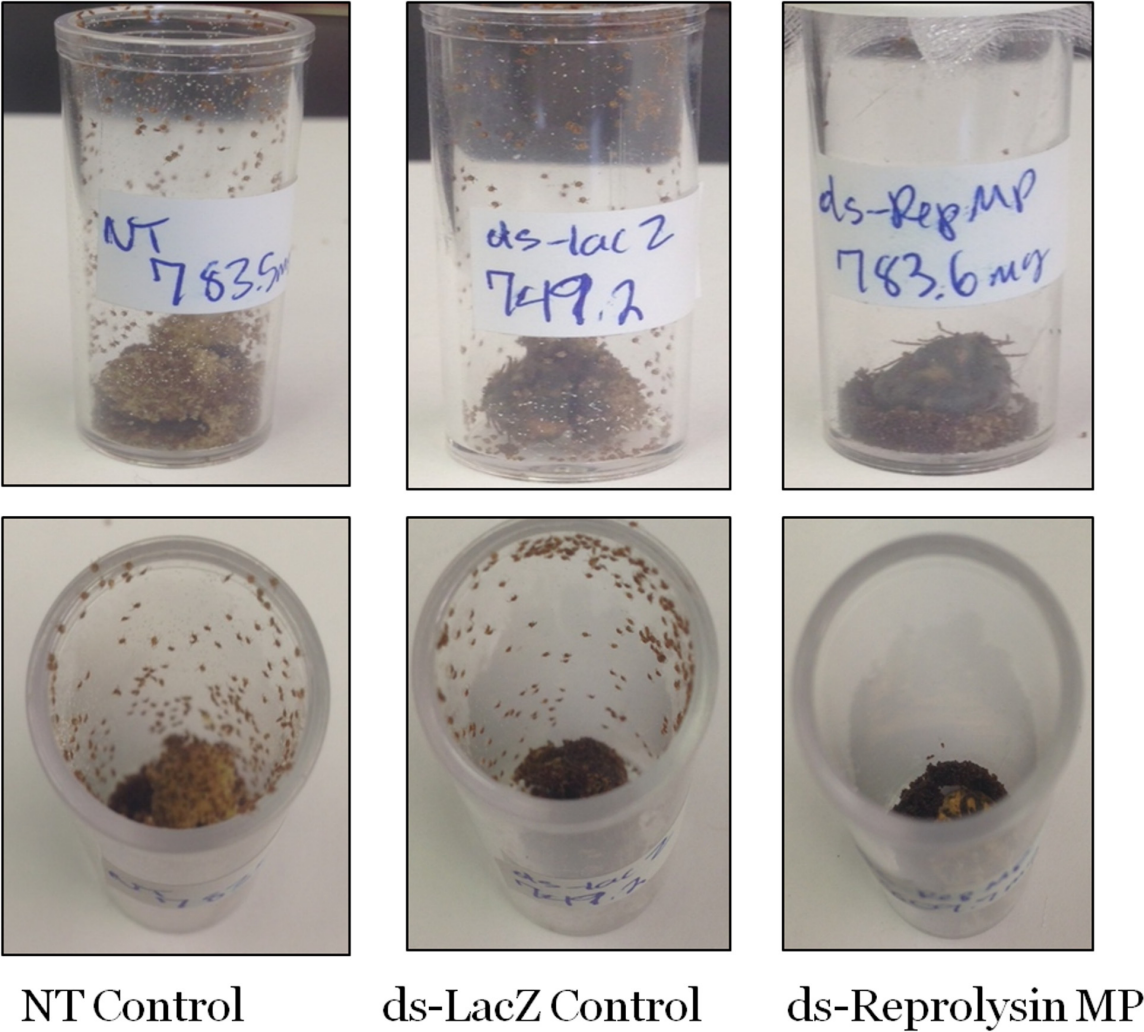
Larval hatching of tick eggs after ds-Reprolysin multiple gene knockdown. Depletion of MP transcripts negatively affected the hatching of larvae, whereas no changes in the control samples were observed.

Using the high MP sequence similarity to our advantage, multiple reprolysin genes were knocked down (Fig 9). The > 95% depletion of the transcript of four Rep-MPs observed in the dsRNA-Rep-treated salivary glands indicated successful silencing (Fig 10A). The depletion of the reprolysins caused a reduction in the tick engorgement weights, and more than 40% of the dsRNA-Rep-injected ticks weighed less than 200 mg (Fig 11). Egg hatching from the engorged female *A*. *americanum* ticks was also affected, as fewer larvae were able to emerge from the egg masses of the dsRNA-Rep-knockdown ticks (Fig 12). To knock down the single MP gene, we targeted a region of the gene with less homology to other MP genes than the region used to knock down the gene family. However, it is noteworthy that the level of homology present within the family was high enough to cause “off-target” effects, preventing the proper testing of each individual MP.

Previous work using salivary gland MPs as vaccine targets reduced the ovipositioning efficiency and adult female weight gain in the ticks. Recently, a transcriptional network has been demonstrated between the two groups of metzincin MPs (reprolysin and astacins) [63]. Therefore, it is possible that despite the completion of feeding by the Rep family knockdowns, a reduction in transcriptional networking impaired their oogenesis. It should also be noted that, because of the sequence identity among the remaining *A*. *americanum* salivary reprolysin metalloproteases, the group knockdown probably affected an additional 5–8 transcripts, potentially depleting over 30% of the total MP transcripts (data not shown). Therefore, it is possible that the MPs present in the ovaries were affected by the dsRNA, which would explain the effects observed on larval hatching.

#### Compensatory Mechanisms for MP Knockdown

Many MP sequences have been identified within the sialotranscriptome. We hypothesized that MPs not affected by family knockdown would be upregulated in response to the depletion of the four MP transcripts. Real-time PCR studies showed a three-fold upregulation of Aam-20700 in response to multigene RNAi knockdown (Fig 10A). Neprolysin MPs were assessed for their roles in any compensatory mechanisms. It has previously been reported that unsilenced metzincin transcripts are upregulated in response to the silencing of reprolysin transcripts [63]. This suggests a potential network between reprolysin MPs and another class of MPs, the astacins, which are more commonly expressed in the ovaries. The redundancy of the tick genome provides many alternate proteins to replace depleted proteins by switching to the expression of redundant genes. The presence of these compensatory mechanisms is probably responsible for the incomplete phenotype observed in the MP-knockdown ticks. The reduced weight of the ticks after multigene knockdown suggests that these ticks are less able to access the host blood because the lack of MPs in their saliva alters the formation of the blood pool. The changes in expression of individual genes after the multigene knockdown may not follow the same pattern, which may be responsible for the differing levels of compensatory gene expression.

#### 16S Bacterial Load

**Fig 13:**
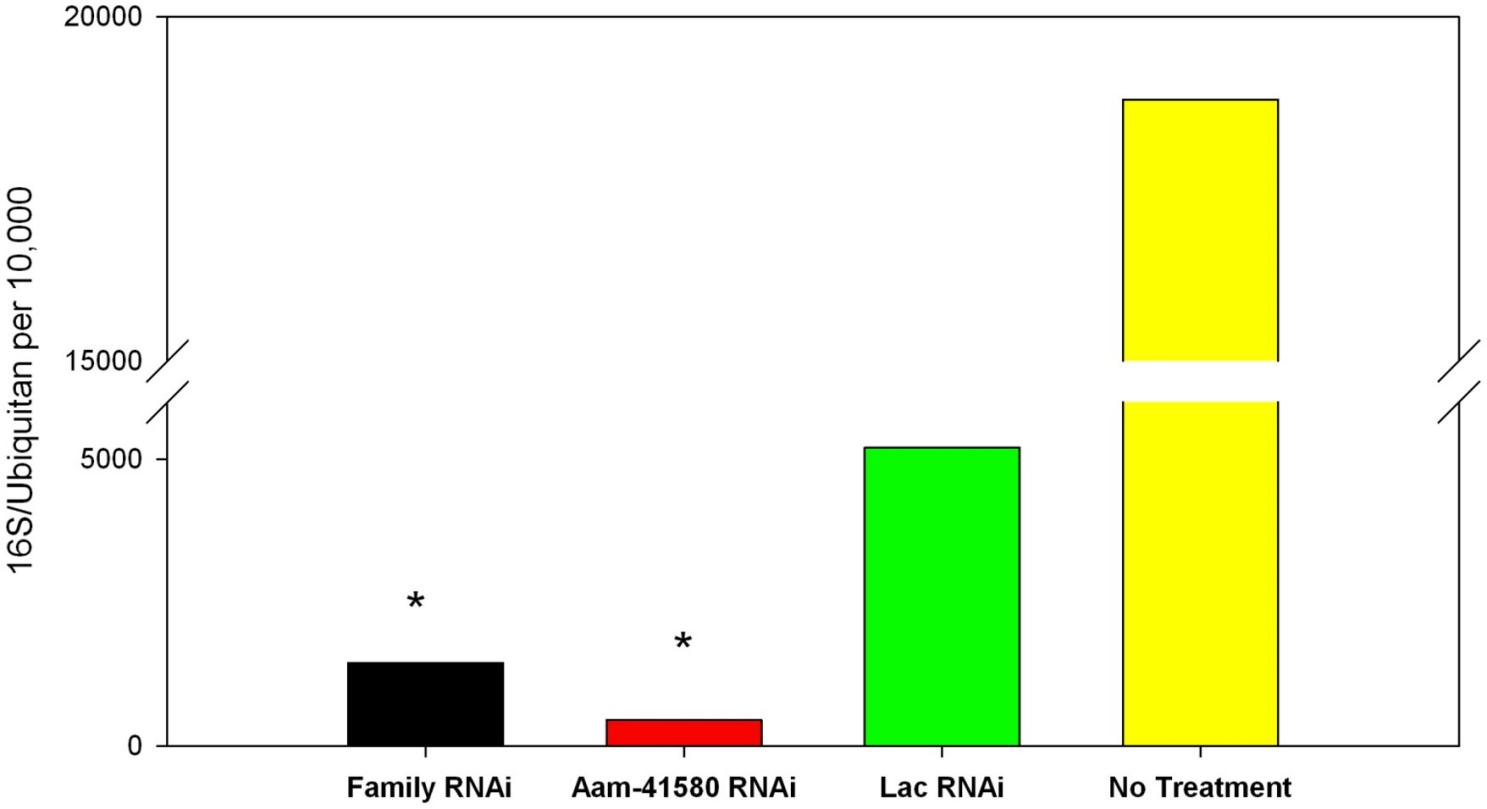
Analysis of microbial load in female salivary glands. The salivary glands of ticks treated with both Rep-Family RNAi and Aam-41580 RNAi were assessed for their total bacterial loads on day 8 of feeding. MP depletion in the salivary glands caused a significant reduction in the total microbial load in ticks in which both the MP family and single genes were knocked down. Bacterial loads after both types of knockdown were 5–12-fold lower than those of the Lac-Z-RNAi-treated and no-treatment control salivary glands. *P < 0.05.

A qRT–PCR analysis of the 16S rRNA in the salivary glands of both RNAi knockdowns showed significant reductions in the 16S bacterial load in the reprolysin-knockdown ticks compared with that in the controls (Fig 13). Ticks that were not injected showed bacterial loads reaching 20,000 bacteria for every 10,000 ubiquitin. When the ticks were injected with dsRNA-LacZ as an injection control, the bacterial load dropped to less than 10,000 bacteria per 10,000 ubiquitin. This reduction in bacteria in the presence of an irrelevant dsRNA could be attributable to the stress induced by the injection. However, when MP genes were knocked down (both individual and multigene knockdown), the bacterial load decreased to less than 2,000 bacteria per 10,000 ubiquitin. Tick bacterial symbionts are tissue specific. An example of this spatial specificity is the dominance of *Coxiella* in the bacterial population within the oocytes of female *Rhipicephalus* species and its significant role in successful ovipositioning [70]. Although no clear link between MPs and microbial load was evident, the reduction in bacteria was significant, and therefore warrants further investigation. The assay used here did not specify the bacterial species that were present and were affected. It is possible that the depletion of MPs interfered equally with all species of endosymbionts or that a dominant species was affected, causing a dramatic reduction in the total number of bacteria. Further investigation, including 16S rRNA sequencing, will more exactly define the effects of MPs on the tick microbiome. Understanding the bacterial communities in ticks should extend our understanding of the functions some of these bacterial communities play in the relationship between the host and the vector.

## Materials and Methods

### Ticks and Ethical Statement

Ticks were purchased from the Oklahoma State University Tick Rearing Facility. Adult male and female *A*. *americanum* were kept according to standard practices [71] at room temperature (25°C) with approximately 90% relative humidity under a photoperiod of 14 h light/10 h dark. The ticks were fed on a sheep and approximately 20–25 female ticks were removed at 24 h intervals (24–168 h) throughout the blood meal. All animal experiments were performed according to approved IACUC protocols (10042001) of the University of Southern Mississippi and in strict accordance with the recommendation in the Guide for the Care and Use of Laboratory Animals of the National Institutes of Health, USA.

### Tick Tissue Dissection

Unfed female ticks and partially fed female ticks from different time points (unfed, 24, 48, 72, 96, 120, 144, and 168 h) were removed from the sheep and dissected. The salivary glands and midguts were removed and placed in ice-cold M199 buffer. The tissues were cleaned of contaminating tissue with fresh M199 buffer. The salivary glands and midguts collected at each time point were pooled according to tissue type and stored in RNAlater (Life Technologies, Carlsbad, NM) at –80°C until analysis [72].

### Gene Expression Analysis

#### RNA isolation and cDNA synthesis

Frozen tick tissues were placed on ice to thaw and the RNAlater was carefully removed with precision pipetting. RNA was isolated from the time-point-pooled salivary glands and midguts using the illustra RNAspin Mini RNA Isolation Kit (GE Healthcare Lifesciences), with the manufacturer’s protocol modified for increased RNA concentrations. RNA concentrations were measured with a NanoDrop spectrophotometer and stored at –80°C or used immediately. To synthesize the cDNA, 2 μg of RNA was added to a 20 μl reaction of the iScript cDNA synthesis kit (Bio-Rad, Hercules, CA, USA). The reverse transcription reaction was then heated in a Bio-Rad thermocycler under the following conditions: 5 min at 25°C, 30 min at 42°C, 5 min at 85°C, and hold at 10°C. The resultant cDNA was diluted to a working concentration of 25 ng/μl with nuclease-free water and stored at –20°C until analysis.

#### Analysis of housekeeping genes

Each gene was PCR amplified to verify the product size and the specificity of the primers. The thermocycling conditions for PCR amplification were: 95°C for 5 min; 35 cycles of 95°C for 5 s, and 60°C for 30 s; followed by 72°C for 7 min, and a 10°C hold. The purity and size of each PCR product was analyzed on a 2% agarose gel stained with SYBR® Safe DNA Gel Stain (Invitrogen) and visualized with the GelDoc system (Bio-Rad). The PCR products were purified with the QIAquick PCR Purification Kit (Qiagen, CA). A standard curve was constructed for each gene using the Bio-Rad CFX 96 Real Time System on a C1000 Thermal Cycler under the same thermocycling conditions used for the PCR. Each of the seven genes was then assessed separately in all eight time point samples from the salivary glands and midguts. The data from each of these samples were used to determine the expression stability of the genes with three different algorithms (BestKeeper, NormFinder, and ΔΔCt).

#### Quantitative real-time PCR

A list of all genes tested and their primers is given in S3 Table. qRT–PCR was performed according to the guidelines of the Bio-Rad protocol provided with the iTaq Universal SYBR Green Supermix (Bio-Rad). Briefly, 50 ng of cDNA was added to a 20 μl qRT–PCR reaction using SYBR Green Supermix, with 300 nM of each gene-specific primer. The samples were subjected to the following thermocycling conditions: 95°C for 30 s; 35 cycles of 95°C for 5 s, and 60°C for 30 s, with a fluorescence reading after each cycle; followed by a melting curve from 65°C to 95°C in 0.5°C increments. Each reaction was performed in triplicate, together with a no-template control. Gene expression was normalized with ubiquitin as the reference gene.

### Verification of MP Knockdown and Phenotypic Analysis

#### Synthesis of dsRNA

Using PCR, a DNA template was generated in which a T7 promoter sequence was added to both the 5′ and 3′ ends of each gene-specific PCR product. The genes of interest are listed in S3 Table. First, Aam-41580 was amplified using gene-specific primers without the T7 promoter, PCR purified, and then amplified in a second PCR reaction with the Aam-41580 primers containing the T7 flanking sequences (S3 Table). The PCR product from the second PCR was purified with the QIAquick PCR Purification Kit (Qiagen) and sequenced (Eurofins MWG Operon) to ensure the proper addition of the T7 sequences. Once the sequence was verified, the PCR product was transcribed into RNA using the HiScribe™ T7 Quick High Yield RNA Synthesis Kit (New England Biolabs, Ipswich, MA) by incubating it with T7 polymerase overnight at 37°C. The resultant dsRNA was purified with ethanol precipitation, its concentration measured spectrophotometrically with a NanoDrop spectrophotometer, and analyzed on a 2% agarose gel. The dsRNA was diluted to a working concentration of 500 ng/μl. The same methodology was used to produce the dsRNA for knockdown of the reprolysin family, and the reprolysin primers were designed based on an alignment of four of the five genes identified in the sialotranscriptome. The reprolysin primer sequences are listed in S3 Table. The T7 flanking sequences were added as described above.

#### Injection of dsRNA and phenotypic assessment

Unfed females ticks were injected with 1 μl of dsRNA-Aam41580, dsRNA-RepMP, or dsRNA-LacZ to a final amount of 500 ng, using a 31-gauge needle. Once injected with dsRNA, the ticks were maintained at 37°C overnight under 90% humidity. The following morning, the surviving ticks were placed on an experimentally naïve sheep and allowed to establish a blood meal. Ticks were removed on days 5 or 6, day 8 or 9, and day 12 postinfestation. The replete ticks were monitored for total engorgement weight, survival, ovipositioning, and larval hatching.

### Quantification of Bacterial Load

The bacterial load was estimated as described previously [73], but with modification for *A*. *americanum* [74]. Briefly, primers were designed within the homologous regions of 16S rRNA and used to amplify the 16S rRNA of *Staphylococcus aureus* to construct a standard curve. A standard curve was similarly constructed for ubiquitin. Each qRT–PCR reaction contained 200 nM primers, iTaq Universal SYBR Green Supermix (Bio-Rad), and the serially diluted PCR products. The thermocycling conditions are listed above in the qRT–PCR methods. Using the standard curves, the total copy numbers of 16S rRNA and tick ubiquitin were calculated for each of the 25 ng cDNA samples. The 16S rRNA copy numbers were normalized to those of the tick ubiquitin gene.

### Statistical Analysis

The data are expressed as means ± SEM, and statistical significance across all experimental groups was calculated with Student’s *t* test using SigmaPlot 12.0. P values < 0.05 were considered significant.

## Conclusions

The sialotranscriptome of the Lone Star Tick, *A*. *americanum*, was used here to clarify the temporal expression of 44 selected genes. Before this study, reference genes were primarily selected by comparing their expression in multiple tissues and selecting the housekeeping gene most stably expressed in all tissues. Here, we have shown that the expression profiles of housekeeping genes differ between tissues and we suggest that reference gene selection take into account these differences. We propose that reference genes should be selected for each individual tissue where possible. In this study, the expression of ubiquitin in the salivary glands remained consistent throughout the blood meal and was shown to have the most stable expression profile according to the ΔΔCt and BestKeeper normalization algorithms. In the midgut, the most stably expressed housekeeping gene was histone H3, determined with the same methods.

This detailed expression analysis of *A*. *americanum* proteins across multiple feeding time points extends our understanding of the functions they play in feeding. It is possible that antigenic variations have a role in these differing expression profiles. Because the host immune system recognizes the tick proteins and mounts a response, the tick may reduce the expression of the initial set of proteins and begin to express proteins with similar functions but differing antigenic properties. This switch to multiple sets of proteins may be a unique biological attribute that allows successful blood feeding. A better understanding of the diversity and varying temporal expression of these related salivary protein families should allow for the development of antitick vaccines, including a cocktail approach for more efficient vaccination.

The identification of the tick proteins responsible for successful blood feeding and pathogen transmission should also allow the accurate identification of target molecules for reverse genetic techniques. Here, five members of the MP family were chosen for further analysis. When we knocked down the expression of these genes, we observed differences in the engorgement weights, and egg hatching success of the ticks, and changes in their 16S-rRNA-based microbial loads. Further analyses are ongoing to characterize the roles of these proteins in the feeding success and pathogen transmission of *A*. *americanum*.

## Supporting Information

S1 Table Expression stability of seven candidate reference genes for the tick salivary glands, calculated with Bestkeeper, ∆∆Ct, and NormFinder. (PDF)

S2 Table Expression stability of seven candidate reference genes for the tick midgut, calculated with Bestkeeper, ∆∆Ct values, and NormFinder. (PDF)

S3 Table Classification of genes and their corresponding primers. (PDF)
