# Supplementary material for: Temporal Gene Expression Analysis and RNA Silencing of Single and Multiple Members of Gene Family in the Lone Star Tick Amblyomma americanum
Source: PLoS One. 2016 Feb 12;11(2):e0147966. doi: 10.1371/journal.pone.0147966 (PMC4752215; doi:10.1371/journal.pone.0147966)
Supplement: S1 Table — (PDF) [file pone.0147966.s001.pdf]

Table S1: Expression Stability of seven candidate reference genes for tick salivary glands as calculated by Bestkeeper, ΔΔCt, and NormFinder.

Rank	Bestkeeper		ΔΔCt		NormFinder	
	**Gene**	**SD**	**Gene**	**SD**	**Gene**	**Stability**
1	Ubiquitin	0.27	Ubiquitin	0.22	Actin	0.068
2	Actin	0.53	Actin	1.24	Calrectulin	0.069
3	GAPDH	0.69	Histone H3	1.41	Ubiquitin	0.072
4	Calrectulin	0.96	GAPDH	1.45	HSP	0.134
5	HSP	1.11	Calrectulin	7.18	GST	0.140
6	Histone H3	1.49	HSP	7.27	Histone H3	0.169
7	GST	1.67	GST	20.83		
