# Supplementary material for: Temporal Gene Expression Analysis and RNA Silencing of Single and Multiple Members of Gene Family in the Lone Star Tick Amblyomma americanum
Source: PLoS One. 2016 Feb 12;11(2):e0147966. doi: 10.1371/journal.pone.0147966 (PMC4752215; doi:10.1371/journal.pone.0147966)
Supplement: S2 Table — (PDF) [file pone.0147966.s002.pdf]

Table S2: Expression stability of seven candidate reference genes for tick midguts as calculated by Bestkeeper, ΔΔCt values, and NormFinder.

**Rank**	**Bestkeeper**		**ΔΔCt**		**NormFinder**	
	**Gene**	**SD**	**Gene**	**SD**	**Gene**	**Stability**
1	Histone H3	0.25	Histone H3	0.31	Calrecticulin	0.058
2	GADPH	0.70	GADPH	0.55	HSP	0.088
3	Actin	0.78	Ubiquitin	0.78	Histone H3	0.175
4	Calreticulin	0.86	Calreticulin	1.69	Actin	0.217
5	HSP	0.90	HSP	2.37	GST	0.375
6	Ubiquitin	1.08	Actin	2.60	Ubiquitin	0.570
7	GST	1.11	GST	10.75		
