# Supplementary material for: Temporal Gene Expression Analysis and RNA Silencing of Single and Multiple Members of Gene Family in the Lone Star Tick Amblyomma americanum
Source: PLoS One. 2016 Feb 12;11(2):e0147966. doi: 10.1371/journal.pone.0147966 (PMC4752215; doi:10.1371/journal.pone.0147966)
Supplement: S3 Table — (PDF) [file pone.0147966.s003.pdf]

Table S3: Classification of genes and their respective primers.

Gene Classification	Gene Name	Forward Primer 5'→3'	Reverse Primer 5'→3'
Housekeeping genes	Aa GAPDH	CTTCTTGGCACCTCCATCAA	GGTGCAGAGTTTGTGGTAGAG
	Aa Histone H3 (GI:759084459)	GAAGCCAGTGAGGCATACTT	GCTCGATATCCTTTGGCATGA
	Aa Calrecticulin (GI:3924592)	ACTACAAGGGCAAGAACCACCTCA	TCGATCTGCTTGGGTTTCCACT CA
	Aa GST (GI:196476640)	CTTCCCGAATTTGCCCTACTA	CGTCAAGACCGTGCTTCTTT
	Aa Ubiquitin (GI:759085449)	GCTGTCCGACTACAACATTCA	GGGTGGTGTAGTTCTTCTTCTT
	Aa β-Actin (GI:759085843)	TCCTATCCTCACCCTGAAGTA	ACGCAGCTCGTTGTACAAG
	Aa HSP (GI:196476687)	CTCTTGTACGCAGGCTTCTTA	TATCATGATGCCGCCCTT
Protease Inhibitor Domains	Aam-36184 (GI:759087923)	TCCTATACGCACTGCTCCGT	CGGGGAGTTGATAATATAGCGA
	Aam-41471 (GI:759087921)	ACCTGCTTCCTATACGCCCT	AGTTGTTGTTATTTCCGCCG
	AamerSigP-40989 (GI:759089359)	ATGCAGCTCCTCACGATTTT	CCACCCTTTGTGGTTGGTAG
	Aam-35414 (GI:759088299)	TCATAGCCGTACTCAGCGTG	TTGAGCACAAATCCTCATGC
	AamerSigP-41415 (GI:759088597)	CATGAGACACCCGAGCTGTA	CAAAGGCCGCTTTTAGAGTG
	AamerSigP-26789 (GI:759087891)	CACTCTCAGTGGTTTTCGCA	CCAGAGTTGCCAGCAGTTTT

Lipocalins	Aam-41264 (GI:759089713)	GGCATCAATGGAAGTTGCTT	GTGTGTGAACGGACTGAACG
	Aam-41091 (GI:759089695)	TGAAACTAAACTCCGCTGCC	CATGTTAACCGTGCCACTTG
	AamerSigP-40605 (GI:759089699)	GGCACCAAACGTTCATCTTT	GCCATTCTTTATGCGGACAT
	AamerSigP-12055	GATTGTGTGGAATTGCCTCC	TTGTGTTCAGGCTGTGGTTC
	AamerSigP-33384 (GI:759088385)	TCATTTTTGATGCTCGTTGG	CATATGTTTTGCTCACGTCCA
	Aam-41375 (GI:759089833)	TTCTTTTCAACGGACCCAAG	CTGGTTTTTGGCATCCAACT
	AamerSigP-18604	TCTTGCCATAACCGCTTTTC	AAATCACTTCCGTCACCAGG
Glycine Rich Proteins	Aam-41235 (GI:759088199)	AGCAGCCTCTCCAGTAGTGC	TCTGGAGAGCCCACCAATAC
	AamerSigP-34358 (GI:759089759)	TGCGAGGAGAAAGTGGAAGT	ATAATGGGACCAGGGTTTCC
	Aam-40766 (GI:759090089)	GATTTGGTGGCTCACTTGGT	AAGACCAGTGCCAAATCCAC
	AamerSigP-39259	AGACTTTCTTGGCATGCTCC	ACTTCCACTTTCTCCTGGCA
	AamerSigP-41913 (GI:759088551)	GTGGAGCTGAAACCTCTGTTAG	TCTGGAGTCTCACCTTCATCTC
	AamerSigP-41539 (GI:759088517)	GCTAGATGACGCTGGGTTT	TCCAAGGTTTCCAGAGTTGTAG
	Aam-41540 (GI:759086339)	GGGAAATCTTGGCGGTATCT	GCCTCCATACCATCCGTTAAA
	Aam-36909	AGCGGTGGATACAGTTTCTATG	TCCAAGACCAGTGCCAAATC

	(GI:759090119)		
	Aam-3099<br>(GI:759089715)	GCCTTCTGTCAGCTGTATGT	CGGCGTAGAGATTACCCTTTC
Tick Specific Genes (Unknown Function)	AamerSigP-40930	ATGAATGCGGCAAAGCTG	TATGGCTCCGGCTGTGTACT
	AamerSigP-41354	GCAATGATGGTCTTTCGGTC	TCTGCAGTAGCCTGGTCCTC
	AamerSigP-41425	CACTTCTCTGCGTGCTTCTG	CTGAATCATTGCTTCTGCGA
	AamerSigP-35954<br>(GI:759088829)	TTAATACAGCAAATCACCGCC	TACCGGTGATAGATGCCTCC
	AamerSigP-15297<br>(GI:759089247)	GGCATCAATGGAAGTTGCTT	GTGTGTGAACGGACTGAACG
	AamerSigP-39321	AGCTGCTGTCATTCACCCTT	ATCCATGTTGTGTGCCGTC
	AamerSigP-22563	TGAATGTCGCTTTGTTCTGC	GTTTGCATCTGACCTCCCAT
Immunity Related Proteins	Aam-12127<br>(GI:759085065)	AGACGAACTAAACGCAAGGG	GTTTTTGTTTAGGAACGCGG
	Aam-31196	TTCAGATGGTGACCCAAACA	AAAGCACACGCTTTCATCCT
	Aam-6020<br>(GI:759087765)	GTTATCTGGTGGTTGCGGTC	ACCATGGATTATTGTTGCCG
	Aam-40185<br>(GI:759087775)	ACGTCCTCTGGTATATGCGG	CCACCAATGTTCTCCTACGC
	AamerSigP-41992	TCAAGTCGTGCGTTACCTTG	CGTGATGTTTCGGTGAGCTA
Other Proteins	Aam-22013	TGTTCTCCCCATCAACATCA	TTTTGCCTTCAAGGTGCTTC

	Aam-40687 (GI:759087721)	GCGAGCAAGAAAAGGATGAC	TTCGACGAAGCAATCAACAG
	AamerSigP-25878 (GI:759089279)	TACGAGCTCTCGAGGTCCAT	GGCATTTCCCCAATCTATCA
	Aam-5252	GGCTGAGATCGAGAAATTCG	CCTGCTTGCTTCTCCTGTTC
Metalloprotease s	AamerSigP-41953 (GI:759090217)	GATGCCAGCCAAGAACAGAT	TGCCAGTCAACTGTTTCCAG
	AamerSigP-41680 (GI:759090211)	GACGCTCAACTTGGAAGGAG	GTTGCGCTCAATCTCGTGTA
	AamerSigP-20700 (GI:759088021)	CTCTGCTTTTTGCAGCTGTG	CTCGTTTCCGTCATACAGCA
	Aam-41579 (GI:759090035)	TGGAATCGAGGATATCAGCC	TTTGTCCATGCCAGTAACCA
	AamerSigP-35996 (GI:759090059)	GACGCTCAACTTGGAAGGAG	TCATTTCGCTCAATCTCGTG
	Aam-41580 (GI:759086485)	ACGAAGAAAAGATCGCCTCA	TGGCTGATATCCTCGATTCC
Other Primers	Reprolysin Family	CACGATGACCTGACGCTCAA	GCTCAATCTCGTGTATCA
	T7 Flanking	GAATTAATACGACTCACTATAGGG AGAACGATCACCTGACGCTCAA	GCTCAATCTCGTGTATCAGAAT TAATACGACTCACTATAGGGAG A
	16S rRNA	AGAGTTTGATCCTGGCTCAG	CATGCTGCCTCCCGTAGGAGT
